# Low-Level Laser Stimulation on Adipose-Tissue-Derived Stem Cell Treatments for Focal Cerebral Ischemia in Rats

**DOI:** 10.1155/2013/594906

**Published:** 2013-12-02

**Authors:** Chiung-Chyi Shen, Yi-Chin Yang, Ming-Tsang Chiao, Shiuh-Chuan Chan, Bai-Shuan Liu

**Affiliations:** ^1^Department of Neurosurgery, Taichung Veterans General Hospital, Taichung 40705, Taiwan; ^2^Department of Physical Therapy, Hung Kuang University, Taichung 43302, Taiwan; ^3^Department of Medicine, National Defense Medical Center, Taipei 114, Taiwan; ^4^Tri-Service General Hospital, National Defense Medical Center, Taipei 114, Taiwan; ^5^Graduate Institute of Pharmaceutical Science and Technology, Central Taiwan University of Science and Technology, Taichung 40601, Taiwan; ^6^Department of Medical Imaging and Radiological Sciences, Central Taiwan University of Science and Technology, Taichung 40601, Taiwan

## Abstract

This study investigated the effects of large-area irradiation from a low-level laser on the proliferation and differentiation of i-ADSCs in neuronal cells. MTT assays indicated no significant difference between the amount of cells with (LS+) and without (LS−) laser treatment (*P* > 0.05). However, immunofluorescent staining and western blot analysis results indicated a significant increase in the neural stem-cell marker, nestin, following exposure to low-level laser irradiation (*P* < 0.05). Furthermore, stem cell implantation was applied to treat rats suffering from stroke. At 28 days posttreatment, the motor functions of the rats treated using i-ADSCs (LS+) did not differ greatly from those in the sham group and HE-stained brain tissue samples exhibited near-complete recovery with nearly no brain tissue damage. However, the motor functions of the rats treated using i-ADSCs (LS−) remained somewhat dysfunctional and tissue displayed necrotic scarring and voids. The western blot analysis also revealed significant expression of oligo-2 in the rats treated using i-ADSCs (LS+) as well as in the sham group (*P* < 0.05). The results demonstrated that low-level laser irradiation exerts a positive effect on the differentiation of i-ADSCs and can be employed to treat rats suffering from ischemic stroke to regain motor functions.

## 1. Introduction

Stroke has become a common disease and has been shown to be associated with the consumption of high amounts of oil and salt. These dietary habits cause blood vessels to narrow and become prone to occlusion, which can lead to stroke. Strokes can be broadly classified into 2 categories: ischemic and hemorrhagic strokes. Differing treatment methods are used according to stroke type and lesion location. Therefore, at stroke onset, computed tomography or magnetic resonance imaging is often used for diagnosis to provide physicians with a basis of treatment [1]. The treatment methods used for the 2 types of stroke are different. (1) Ischemic stroke is caused by thrombosis or blood clots in the brain vessels, which prevent blood flow into the brain tissues. Anticoagulants or antiplatelet medications must be administered to patients as soon as possible. (2) Hemorrhagic stroke is caused by the rupture or hemorrhaging of brain vessels caused by peripheral brain trauma (subarachnoid hemorrhage). The majority of cases of hemorrhagic stroke are caused by brain trauma (e.g., car and workplace accidents). Treating these patients often requires neurosurgical interventions [2]. Ischemic strokes constitute the majority of stroke cases. When patients suffer from ischemic stroke, the brain tissues necrotize gradually because of the lack of nutrients if anticoagulants or antiplatelet medications are not administered within 3 h of the stroke. After necrosis occurs, necrotic brain tissue cannot regenerate or recover its functions even if blood-vessel reperfusion occurs [3]. In recent years, stem cell treatment has become an innovative therapy for brain tissues that cannot regenerate.

Body fat extraction (e.g., abdomen, thigh, or buttock fat) has been used for body shaping procedures in the past, and the extracted fat was discarded as medical waste. Presently, medical experts have confirmed that adipose tissues contain large amounts of mesenchymal stem cells that exhibit in vitro proliferation and multiple differentiation ability, characteristics that facilitate the repair and regeneration of damaged tissues or organs [4]. In addition, adipose-derived stem cells (ADSCs) possess several characteristics: (1) they can be easily harvested in abundant quantities without invasiveness, (2) they can proliferate in in vitro cultures, and (3) they can be applied to a wide range of body tissue types because these cells migrate to lesion sites automatically to repair damage. Studies have shown that ADSCs can differentiate into many different cell types, such as adipose, bone, cartilage, smooth muscle, cardiac muscle, endothelial, blood, liver, and even neuronal cells [5]. Because of these characteristics, ADSCs will likely be one of the major sources of autologous stem cells in the future.

The effectiveness of laser therapy on biological bodies has been confirmed, and laser therapy has been successfully applied in new technological applications such as microsurgery. Lasers can be classified as high or low power, depending on the energy levels used. High-power lasers involve using high energy levels to provoke blood coagulation, stop bleeding, cut tissues, and even damage cells, whereas low-level lasers involve using electromagnetic or photochemical processes to achieve therapeutic effects [6]. A low-level laser is defined as a laser with extremely low power and energy too low to destroy the molecular bonding capacity (e.g., hydrogen bonds and van der Waals forces) of tissues. Therefore, low-level lasers do not cause molecular structural change, protein denaturation, or cell death. Irradiation from low-level lasers on tissue does not cause an obvious temperature increase at the treatment site (less than 0.1 to 0.5°C); thus, any physiological responses of the tissues are produced by the stimulation of the laser itself, and this mechanism is described by the theory of laser biostimulation [7]. In addition, by using an appropriate energy level, low-level lasers are primarily used to stimulate biological cells to induce or strengthen physiological responses for facilitating local blood circulation, regulating cell functions, promoting immunological functions, and facilitating cell metabolism and proliferation. Using lasers to generate these physiological changes enables treatment goals such as anti-inflammation and wound-healing promotion to be met [8, 9]. Many studies have shown that low-level laser irradiation exerts beneficial biological effects on bone, neuronal, and skin healing [10–12]. However, the type, wavelength, power, and energy level of lasers used in previous studies have varied, and various effects have been observed in different cells when differing levels of laser energy were applied. Previous studies have shown that using a low-level laser with an 820–830 nm wavelength can reduce neural damage, facilitate neuronal healing, and accelerate neural recovery after an osteotomy [13, 14]. Using a low-level laser with a 660 nm wavelength has been demonstrated to exert healing effects on musculoskeletal injuries and inflammation [15]. In addition, many studies have indicated that a low-level laser with a 660 nm wavelength can effectively promote neural regeneration and accelerate the reinnervation of muscle fibers to promote the recovery of motor functions [16–18]. Recent evidence has suggested that protein aggregates such as *β*-amyloid- (A*β*) associated neurotoxicity and dendrite atrophy might be a consequence of brain-derived neurotrophic-factor (BDNF) deficiency. Meng et al. observed that the upregulation of BDNF caused by using low-level laser therapy (LLLT) to activate the extracellular signal-regulated kinase (ERK)/cAMP response element-binding protein (CREB) pathway can ameliorate A*β*-induced neuron loss and dendritic atrophy, thus identifying a novel pathway through which LLLT protects against A*β*-induced neurotoxicity [19].

Currently, most low-level laser therapies in practical clinical applications emphasize treatment courses covering an extensive tissue area in a relatively short period. Multichannel-laser hair treatment, which is currently available for the physical treatment of alopecia, is one such example. Therefore, we used a large-area LLLT that differs from the irradiation methods previously reported in the literature (such as single-spot low-energy laser exposure or the scanning method) to increase the local area of exposure [18]. In this study, stem cells were extracted from adipose tissues, and neural-stem-cell- (NSC-) differentiating agents were used to culture these ADSCs to transform them into induced adipose-derived stem cells (i-ADSCs), which were used as experimental cells. First, we investigated the effects of large-area irradiation from a low-level laser on the proliferation and differentiation of i-ADSCs into neuronal cells. We then investigated whether i-ADSCs treated with laser irradiation and injected via an intravenous route could integrate and survive in various locations in rat brains. We investigated if this treatment could improve the neurological dysfunctions caused by ischemic brain damage in rats and if rats could produce BDNF, using this treatment. Studies using intravenous injection to transplant i-ADSC for the treatment of ischemic stroke with protocols similar to that used in the current study are rare. In this study, we further combined the biostimulation theory from large-area LLLT with transplantation of i-ADSCs to induce neural differentiation to investigate the effectiveness of ischemic stroke treatment. In a previous study [20], we established a transient ischemia-reperfusion stroke rodent model by using right-sided middle cerebral artery occlusion (MCAO) to simulate acute clinical insults. The effectiveness of the treatment was assessed by comparing HE-staining and western blot analysis, as well as the evaluation of motor skill indices by using the rotarod and grip-strength tests. Our protocol has the potential to be developed for application in the clinical treatment of patients with ischemic stroke.

## 2. Materials and Methods

### 2.1. Isolation and Culture of ADSCs

**Figure 1:**
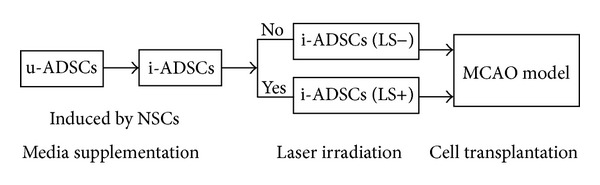
A flowchart illustrating the experimental design. Detailed procedures are described in Section 2.

A flowchart illustrating the experimental design of the study is shown in Figure 1. Eight-week-old male Sprague-Dawley (SD) rats were used for isolating rat ADSCs. The ADSCs were harvested from the rats' subcutaneous anterior abdominal wall. Inguinal fat pads were excised, washed sequentially in serial dilutions of betadine, and finely minced as tissues in phosphate-buffered saline (PBS). The tissues were digested with 0.3% of Type I collagenase (Sigma) at 37°C for 60 min. The digested tissue/cell suspension was filtered through a 100-mesh filter to remove the debris, and the filtrate was centrifuged at 1000 rpm for 10 min. The cellular pellet was resuspended using DMEM/F12 (10% FBS, 1% P/S) and then cultured for 24 h at 37°C in 5% CO_2_. Unattached cells and debris were then removed and the adherent cells were cultured using fresh medium. The cells were cultured to 80% confluency before being released with 0.05% trypsin and then subcultured.

### 2.2. ADSC Neuronal Predifferentiation

In this study, i-ADSCs obtained from culturing ADSCs by adding NSC-differentiating agents were used as experimental cells. ADSCs within 3–5 passages were detached and induced using NSC media supplementation. ADSCs were resuspended in a serum-free DMEM/F12 medium supplemented with an N2 supplement (Sigma), 20 ng/mL of epidermal growth factor (Gibco, NY, USA), and fibroblast growth factor (Gibco, NY, USA).

### 2.3. Setup of a Low-Level Laser Application Method

The probe of the laser irradiation device was fixed vertically on a clean, open experimental bench. The distance between the probe and the cell culture dish was 30 cm. Laser irradiation was applied in a 25°C environment by using an AlGaInP-diode laser (Konftec Co., Taipei, Taiwan) with a wavelength of 660 nm at an output power of 50 mW and frequency of 50 Hz. In the control group, the cells that did not receive laser irradiation treatment, i-ADSCs (LS−) (*n* = 10), were compared with the experimental cells, i-ADSCs (LS+) (*n* = 10), which were subjected to a laser irradiation treatment of 10 min. The cells in the i-ADSCs (LS−) group were cultured for 7 days, whereas the cells in the i-ADSCs (LS+) group were treated using low-level lasers on the following day for 10 min and then cultured for 6 days. The cells receiving laser irradiation were collected at various times for analysis according to the purposes of the experimental protocols. After the completion of the cultures, an optical microscope was used for observing cell morphology.

### 2.4. MTT Assay

The principle of the MTT (3-[4,5-dimethylthiazol-2-y1]-2,4-diphenyltetrazolium bromide) assay is that the mitochondria of living cells can transform the yellow chemical substance MTT tetrazolium into the purple non-water-soluble substance MTT formazan through the effect of succinate dehydrogenase. DMSO can be used to dissolve the purple-colored products. No such response occurs in dead cells. An optical absorbance of 570 nm was measured using an enzyme immunoassay analyzer. A higher absorbance value indicates a larger amount of cells. In this study, ADSCs neuronal predifferentiation was first distributed in a 96-well plate with approximately 10000 cells per well. The cells in the i-ADSCs (LS−) group were then cultured in a 37°C environment and a 5% CO_2_ environment for 5 days and 7 days, respectively. The cells in the i-ADSCs (LS+) group were cultured in a 37°C environment and a 5% CO_2_ environment for 4 days and 6 days, respectively. After the completion of the cultures, the medium was removed by several rinses with PBS. An MTT solution of 100 *μ*L was added to each well of a 96-well plate in the dark (1 mL of MTT reagent was added to 9 mL of phenol-red-free, serum-free medium) and incubated in a 37°C, 5% CO_2_ environment for 2 h. The MTT solution was then removed and the cells were dissolved using DMSO. An optical absorbance of 570 nm was measured using an enzyme immunoassay analyzer to compare the values between the different groups.

### 2.5. Immunocytochemistry of i-ADSCs

After 7 days in culture, the subcultured neurospheres were washed using 0.1 M PBS 3 times and fixed with 4% paraformaldehyde for 1 h. Following the fixation, the cells were permeated with 0.1% of Triton X-100 for 10 min and then blocked with 5% nonfat milk for 30 min. The phenotypic expression of these neurospheres was examined by implementing immunocytochemical staining accompanied by antibodies against glial fibrillary acidic proteins (GFAPs) for astrocytes, mouse monoclonal antinestins for NSCs, and doublecortin (DCX), which has recently been used as a marker for neurogenesis. Briefly, the fixed cells were washed 3 times in cold PBS. After washing with PBS, the aforementioned primary antibodies were added and the slides were maintained at room temperature overnight. In the following day, the primary antibodies were removed by washing 3 times with PBS and the secondary antibodies were added before incubating the cells for 1 h. After washing off the secondary antibodies, the cells were incubated with tertiary antibodies tagged with peroxidase-antiperoxidase for 1 h. The tertiary antibodies were washed off using PBS. The cells were incubated with DAPI (Sigma, St. Louis, MO, USA) diluted with the cell culture medium for 10 min. Finally, the cells were mounted with 90% glycerol and examined using fluorescent microscopy (Olympus IX-71, Inc., Trenton, NJ, USA).

### 2.6. Animals and Induction of the MCAO Model

In a previous study [20], we established a transient ischemia-reperfusion stroke rodent model, using right-sided middle cerebral artery occlusion (MCAO) to simulate acute clinical insults. All of the experimental procedures were approved by the Institutional Animal Care and Use Committee of Taichung Veteran General Hospital, Taiwan. Thirty-two adult male SD rats were randomly allocated to 3 groups: the i-ADSCs (LS−) therapy group (*n* = 12), the i-ADSCs (LS+) therapy group (*n* = 12), and a sham group (*n* = 8). The rats in all 3 groups were euthanized on the 28th day after MCAO was performed. For MCAO procedures, anesthesia was induced using 4% isoflurane (Baxter, USA) and maintained using 2% isoflurane. A midline cervical incision was made to isolate the right bilateral common carotid artery. A 25 mm-long 3-0 nylon surgical thread was then inserted into the right carotid bifurcation. In this study, 2 rounds were used to provide a more complete blockage of blood flow in the artery. When the blunted distal end met resistance, the proximal end of the thread was tightened at the carotid bifurcation. The right common, internal, and external carotid arteries were carefully separated from the adjacent vagal nerve, and the distal portions of the external and common carotid arteries were ligated. A small incision was subsequently made at the proximal portion of the external carotid artery, and a 3-0 nylon monofilament suture was gently inserted (approximately 18 mm) into the internal carotid artery. After 60 min of MCAO, the nylon surgical thread was removed to allow complete reperfusion of the ischemic area. During ischemia, rectal temperature was monitored and maintained at approximately 37°C by using a heating pad and an overhead lamp. The anesthetized rats intravenously received i-ADSCs at a concentration of 2 × 10^7^ mL^−1^ via their femoral veins. The rats in the sham group underwent the same surgical procedures except that the right-sided middle cerebral artery was not occluded.

### 2.7. Rotarod Test

An accelerating rotarod test was performed for each rat before and on the 7th, 14th, 21st, and 28th day after cerebral ischemia-reperfusion was induced. Before the ischemia-reperfusion experiment was conducted, the animals were subjected to 3 training sessions per day for 3 days on the accelerating rotarod to obtain stable duration on the rotarod spindle. The diameter of the rotarod spindle was 7 cm. The surface of the rotarod spindle was made of knurled Perspex to provide an adequate grip, which prevented animals from slipping off the spindle. The speed of the spindle was increased from 4 to 40 rpm over a period of 5 min and the duration that the animal stayed on the device was recorded. The rats that were capable of staying on the rotarod longer than 150 s after 3 training sessions were selected for the experiments. On the testing days, the animals were tested twice, and the longest durations on the rotarod were recorded.

### 2.8. Grip Strength Test

Each rat was supported in a horizontal position approximately parallel to a grip bar (Model DPS-5R: range 0–5 kgf, Japan). The researcher set the rat's forepaws on the grip bar and pulled the animal horizontally away from the bar by the base of its tail until the rat released its grip. The pulling motion was smooth and continuous. The researcher supported the rat by the abdomen when the grip was released. The reading on the strain gauge remained constant at the point of maximal value, which was recorded as the measure of forepaw grip strength. The researcher supported the rat body by both the chest and the base of the tail at an angle of −45° down the tail. The rat was facing away from the grip bar. The rat was encouraged to grasp the bar by moving its hind paw to the bar. When the rat grasped the bar with both hind paws, establishing a “full” grip, the upper body of the rat was lowered so that the rat was in a nearly horizontal position. The rat was pulled horizontally by the base of the tail until it released its grip and was supported as previously described. The reading on the strain gauge remained constant at the point of maximal value (force was measured in grams), which was recorded as the measurement of forepaw grip strength. Three values were obtained in succession, and the median value was used as the daily score. The data were expressed as the percentage of the baseline (preischemic) value.

### 2.9. Hematoxylin-Eosin Staining of the Cerebellum

The SD rats were anesthetized using 10% chloral hydrate (4 *μ*L/kg), administered intraperitoneally, and were euthanized on the 28th day after the MCAO operation and sham treatment. For each rat, the left cerebellum was rapidly removed and postfixed in formalin for 24 h. The postfixed tissues were embedded in paraffin wax and 6-*μ*m-thick serial coronal sections were obtained and mounted on poly-L-lysine-coated glass slices. To assess the histological changes in the MCAO and sham groups, the paraffin-embedded left cerebellum sections were stained using hematoxylin-eosin (HE), according to standard protocol before the assay was performed.

### 2.10. Western Blot Analysis

Proteins were extracted from the rat brains by using a cold lysis buffer (10 mM of tetra sodium pyrophosphate, 20 mM of Hepes, 1% Triton X-100, 100 mM NaCl, 2 *μ*g/mL of aprotinin, 2 *μ*g/mL of leupeptin, and 100 *μ*g/mL of phenylmethylsulfonyl fluoride). The protein concentrations from tissue extracts or ADSC-conditioned medium were determined using the Bradford protein assay. Equal amounts of protein were placed in a 2× sample buffer (0.125 M Tris-HCl, pH 6.8, 2% glycerol, 0.2 mg/mL of bromophenol blue dye, 2% SDS, and 10% *β*-mercaptoethanol) and electrophoresed through 10% SDS-polyacrylamide gel. The proteins were then transferred onto a nitrocellulose membrane by using electroblotting. The membranes were blocked for 1 h at room temperature in a Tris-buffered saline with Tween-20 (TBST) and 5% nonfat milk. The primary antibodies (1 : 1000) with appropriate dilutions were incubated for 1 h at room temperature in TBST and 5% nonfat milk. The blots were then washed and incubated with a peroxidase-conjugated secondary antibody (1 : 2000) for 1 h in TBST. The chemiluminescent substrate for the secondary antibody was developed using the ECL detection system (Amersham, UK). The blots were exposed to film for 3–5 min and then developed.

### 2.11. Statistical Analysis

The data were expressed as the mean value ± standard error of the mean. The statistical significance of the differences between the groups was determined using a one-way analysis of variance followed by Tukey's test. An alpha level of less than 0.05 (*P* < 0.05) was considered statistically significant.

## 3. Results

### 3.1. Effects of the Low-Level Laser on Cell Morphology

**Figure 2:**
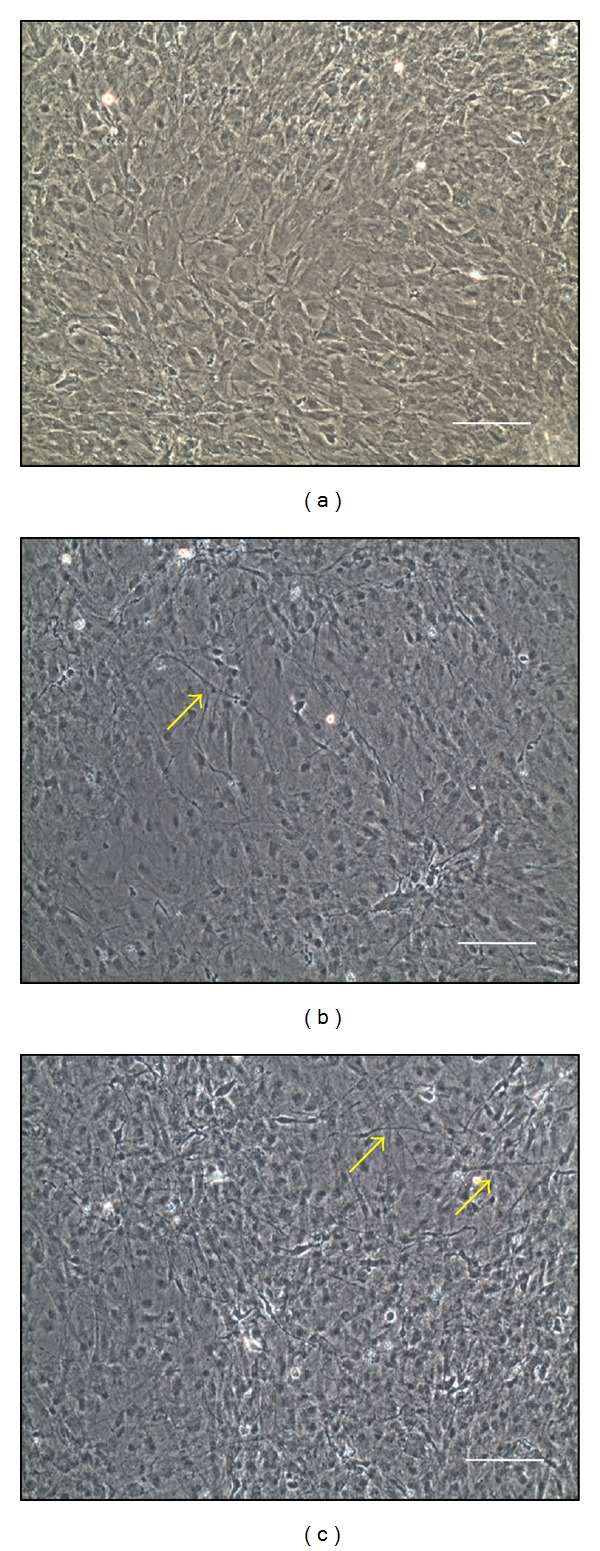
The morphology of inductions of adipose-derived stem cell (i-ADSC) differentiation into neuronal cells after low-level laser irradiation. (a) Undifferentiated ADSCs; (b) i-ADSCs (LS−); (c) i-ADSCs (LS+). The arrow denotes the neuronal-like cells. The scale bar represents 100 *μ*m.

The ADSCs were passaged 3–5 times after the initial plating of the primary culture. Rat ADSCs appeared to be a monolayer of large and flat cells (Figure 2(a)). Many cells in the i-ADSCs (LS−) and i-ADSCs (LS+) groups induced a neuronal phenotype and exhibited, among one another, bipolar and multipolar elongations of neuronally induced cell-forming networks. The results show that the stem cells in both i-ADSCs (LS−) and i-ADSCs (LS+) groups developed tentacles, indicating that ADSCs were facilitating the induction of differentiation into neuronal cells. Comparative optical micrographs revealed that some attached cells exhibited a spread-out shape with a spindle-like and fibroblastic phenotype in the i-ADSCs (LS−) group (Figure 2(b)). However, most of i-ADSCs-expressing neurites extended radially, connecting like bridges with those from adjacent cells in the i-ADSCs (LS+) group (Figure 2(c)).

### 3.2. Effects of the Low-Level Laser on Cell Proliferation and Differentiation

**Figure 3:**
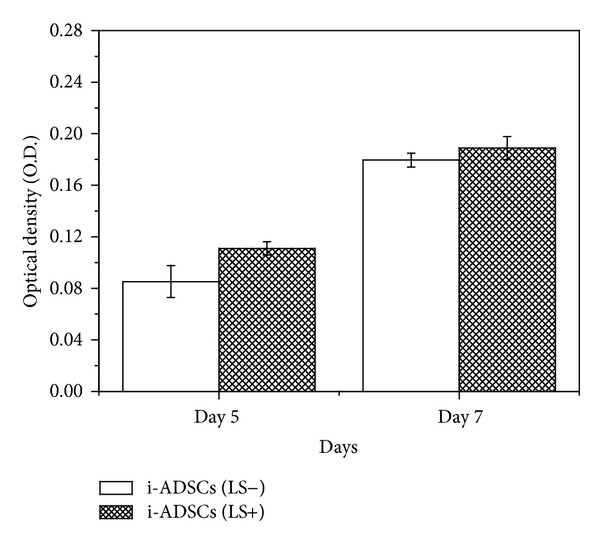
The cell activity of inductions of adipose-derived stem cell (i-ADSC) differentiation into neuronal cells in both the i-ADSCs (LS−) and i-ADSCs (LS+) groups on Days 5 and 7 after culture.

In this study, MTT assays were performed on Day 5 and Day 7 to evaluate the effects of large-area low-level laser irradiation on the facilitation of cell proliferation. After analyzing the optical absorbance values, the results showed that, on Day 5, cell activity was slightly higher in the i-ADSCs (LS+) group compared with that in the i-ADSCs (LS−) group. However, the difference was not statistically significant (*P* > 0.05). On Day 7, the cell amounts in both the i-ADSCs (LS+) and i-ADSCs (LS−) groups were larger than the amounts on Day 5. However, the cell proliferation rates were similar on Day 5 without major differences (*P* > 0.05) (Figure 3).

**Figure 4:**
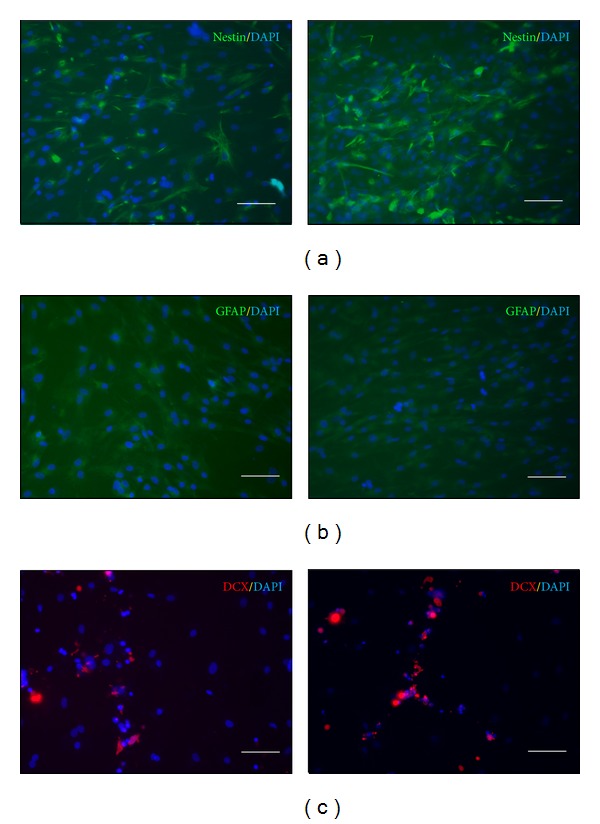
The immunofluorescent staining for (a) nestin; (b) GFAP; and (c) DCX of inductions of adipose-derived stem cell (i-ADSC) differentiation into neuronal cells in both the i-ADSCs (LS−) (left) and i-ADSCs (LS+) (right) groups. The scale bar represents 100 *μ*m.

In this study, immunofluorescent staining and western blots were used to evaluate the effects of large-area low-level laser irradiation on the facilitation of cell differentiation. Immunofluorescent staining was performed for the NSC marker, nestin, glial cell marker, GFAP antibody, and neuronal precursor-cell-marker protein, DCX. After the staining was completed, fluorescent microscopy was used to observe the amount of fluorescence expression of each antibody. The results showed that the fluorescence expression of the nestin was higher in the cells in the i-ADSCs (LS+) group than that in the cells in the i-ADSCs (LS−) group. These results indicated that ADSC differentiation into neuronal cells was facilitated after large-area low-level laser irradiation (Figure 4(a)). For GFAP, no difference was observed in the amount of fluorescence expression of GFAP between the i-ADSCs (LS−) and i-ADSCs (LS+) groups (Figure 4(b)). Furthermore, for DCX, no difference was observed between the 2 groups because the cells in both groups still exhibited stem cell morphology (Figure 4(c)).

**Figure 5:**
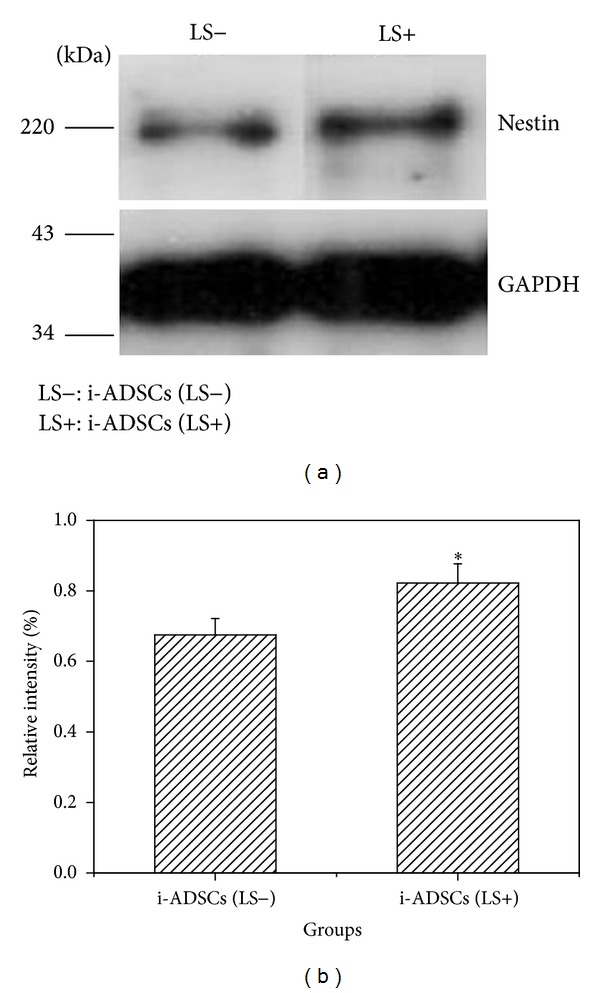
The cell growth of inductions of adipose-derived stem cell (i-ADSC) differentiation into neuronal cells with or without laser irradiation. (a) The amount of nestin expression. (b) The figure showing the quantification. GAPDH served as the internal reference. *Significance (*P* < 0.05) greater than that of the i-ADSCs (LS−) group.

Western blot is used to quantify the expression of marker proteins. Therefore, western blot analysis was used to compare the amount of nestin expression between the i-ADSCs (LS−) and i-ADSCs (LS+) groups in this study. The results showed that cells in the i-ADSCs (LS+) group exhibited a substantially higher nestin expression compared with the cells in the i-ADSCs (LS−) group (*P* < 0.05) (Figure 5). Regarding the results of GFAP and DCX, no difference was observed in the fluorescence expressions of the i-ADSCs (LS−) and i-ADSCs (LS+) groups. Therefore, western blot analyses were not shown for GFAP and DCX. This result is consistent with the findings obtained using immunofluorescent staining.

### 3.3. Evaluation of Behavior Recovery after Stroke in the Animals

**Figure 6:**
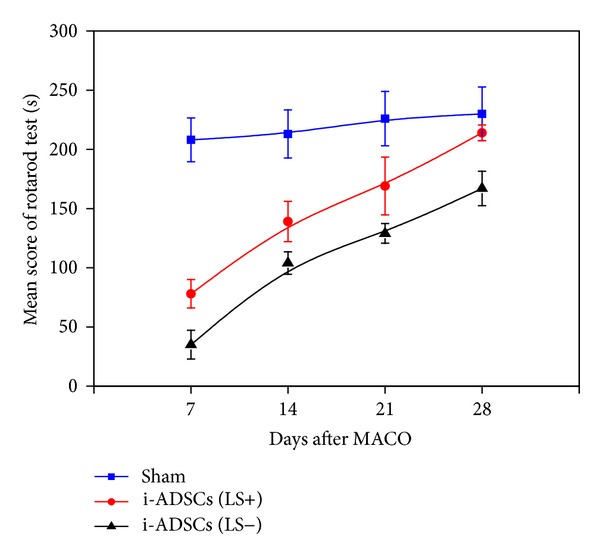
The treadmill test for evaluating the recovery of the motor function of running in rats with ischemic stroke with i-ADSCs (LS−) and i-ADSCs (LS+) transplantation.

In this study, treadmill and forepaw-grip tests were used to evaluate motor function recovery after stem-cell transplantation treatment in rats with ischemic stroke. The treadmill test was performed on Day 7 after stem-cell transplantation was performed on the rats with stroke. The rats from either the i-ADSCs (LS+) group or the i-ADSCs (LS−) group were unable to run as quickly as the rats in the sham group. From Day 14, the rats in the i-ADSCs (LS+) group gradually recovered the ability to run. By contrast, it was observed that the rats in the i-ADSCs (LS−) group recovered slightly; however, the degree of recovery was lower than that in the i-ADSCs (LS+) group. On Day 21 after stem-cell transplantation, the recovery of running function was still more satisfactory in the i-ADSCs (LS+) group than in the i-ADSCs (LS−) group. On Day 28, the motor function of the rats in the i-ADSCs (LS+) group was approaching the level of the rats in the sham group, whereas the performance of the rats in the i-ADSCs (LS−) group was still considerably weaker than that of the sham group (Figure 6).

**Figure 7:**
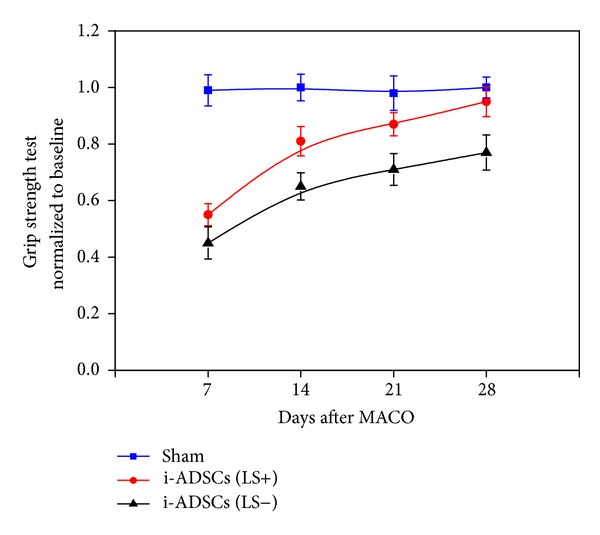
The grip test for evaluating the recovery of grip strength in rats with ischemic stroke treated with i-ADSCs (LS−) and i-ADSCs (LS+) transplantation.

Grip-strength tests were performed on Day 7 after stem-cell transplantation. The findings were similar to the treadmill test results; the grip behavior of the rats in both the i-ADSCs (LS+) group and the i-ADSCs (LS−) group was worse than that of the rats in the sham group. On Day 14, the grip strength of the rats in the i-ADSCs (LS+) group was considerably recovered. By contrast, although the rats in the i-ADSCs (LS−) group showed some progress, the improvement was minimal. On Day 21, the rats in the i-ADSCs (LS+) group continued to recover, whereas it was observed that the animals in the i-ADSCs (LS−) group were not recovering as quickly. On Day 28, grip strength recovery in the i-ADSCs (LS+) group approached that of the sham group, whereas grip strength in the i-ADSCs (LS−) group was still lower (Figure 7).

### 3.4. Repair of Brain Tissues after Treatment in Animals

**Figure 8:**
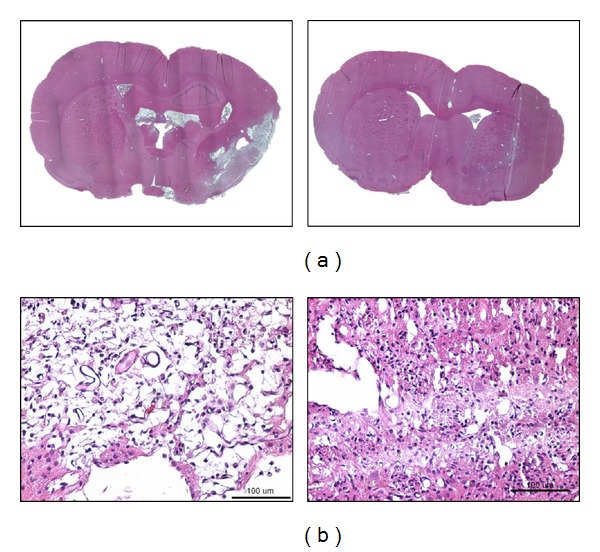
The observation of brain tissue necrosis in rats with ischemic stroke treated with i-ADSCs (LS−) (left) and i-ADSCs (LS+) (right) transplantation: (a) 10x; (b) 200x.

After euthanizing the rats, the brain specimens were treated with paraffin and then sliced. The HE-immunostaining method was used to observe the repair of brain tissues after stem-cell transplantation was performed. An upright microscope (10x) was used to macroscopically observe the brain tissue, and the results showed that brain tissue was completely repaired in the rats in the i-ADSCs (LS+) group with nearly no necrotic brain tissue. By contrast, obvious necrotic scars were observed at the ischemic sites of the brain tissue from the rats in the i-ADSCs (LS−) group (Figure 8(a)). Observed under a microscope and magnified 200 times, the results showed that the brain tissue from the rats in the i-ADSCs (LS+) group was as dense as normal brain tissues, whereas numerous cavities were observed in the brain tissue from rats in the i-ADSCs (LS−) group (Figure 8(b)).

**Figure 9:**
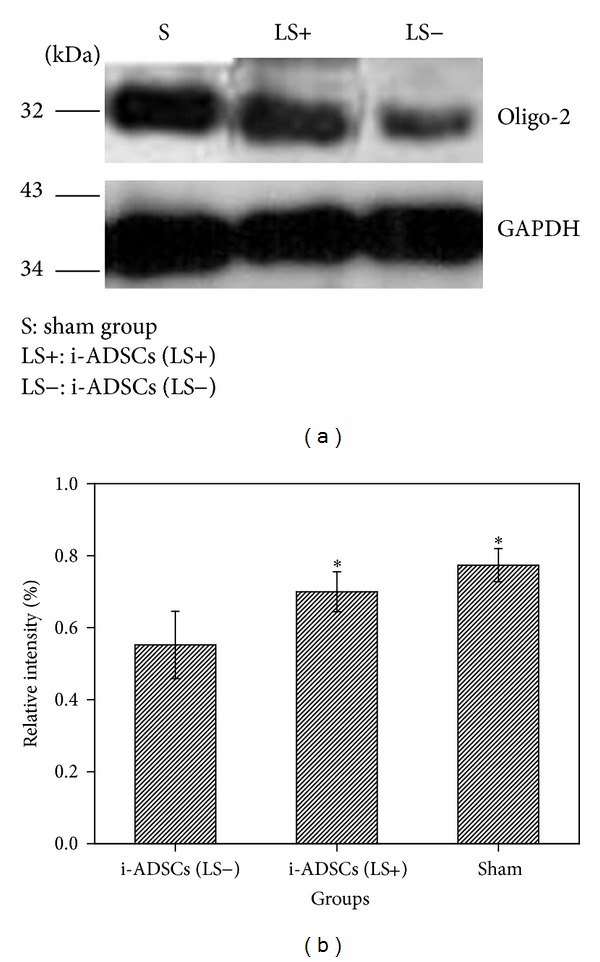
The observation of brain tissue repair in rats with ischemic stroke treated with i-ADSCs (LS−) and i-ADSCs (LS+) transplantation. (a) The amount oligo-2 expression. (b) Quantification. GAPDH served as the internal reference. *Significance (*P* < 0.05) greater than that of the i-ADSCs (LS−) group.

Oligodendrocytes, which are glial cells found in normal brains, form myelin in the central nervous system. Oligodendrocytes substantially decrease after brain cells are damaged, leading to myelin collapse and the loss of neural-signal conduction. Therefore, a western blot was used to analyze the amount of expression of the oligodendrocyte cell protein, oligo-2, to confirm the repair of brain tissues after stem-cell transplantation was performed in rats with stroke. The results showed that the amount of oligo-2 protein response in the brain tissues of the i-ADSCs (LS+) group was as high as that in the sham group. By contrast, the oligo-2 protein response in the i-ADSCs (LS−) group was substantially lower (*P* < 0.05). These results indicated that stem-cell transplantation treatments can repair brain tissues damaged by ischemia (Figure 9).

## 4. Discussion

Stem cells possess the ability to proliferate, regenerate, differentiate, and secrete cytokines. Previous studies have proven that stem-cell therapy substantially improves the damage caused by stroke. Stem cells are derived from various sites. Most previous studies have used bone marrow mesenchymal stem cells; however, we used adipose stem cells in the present study, which are easily accessible, and abundant and exhibit high differentiation and proliferation activity. Moreover, adipose stem cells do not trigger strong immune reactions (resulting in low exclusion) and rarely form teratomas. In the literature, it has been demonstrated that adipose stem cells are essential adult stem cells that differentiate into various mesoderm tissues similarly to bone marrow mesenchymal stem cells [21–23]. Furthermore, adipose stem cells are more easily accessible than mesenchymal stem cells; therefore, adipose stem cells can be used as a substitute for bone marrow mesenchymal stem cells for repairing damaged tissues in the future. Adipose stem cells are generally more practical than bone marrow mesenchymal stem cells for use in research [23–26].

In this study, we used large-area low-level laser irradiation to induce ADSCs to differentiate into neuronal cells. The MTT assay analysis showed that the cell activity of the i-ADSCs increased on Days 5 and 7 of culture after large-area low-level laser irradiation [27, 28]. However, although cell activity increased on both Days 5 and 7, the activity was also increased for the group that did not receive laser irradiation treatment. No significant difference was observed between the groups with or without laser treatment (*P* > 0.05). Previous studies have shown that, depending on irradiation parameters, various types of cell respond differently to laser irradiation [29]. Although the mechanism underlying this phenomenon remains obscure, several hypotheses have been proposed to explain the mechanism of laser action [30]. We speculate that the lack of significant effects might be attributable to the short duration of low-level laser irradiation, which might not provide sufficient energy to the ADSCs for them to facilitate proliferation. Alternatively, using low-level lasers on the ADSCs might not sufficiently enhance cell proliferation. Therefore, future studies should investigate the appropriate duration of low-level laser irradiation and determine how much energy is required to accelerate cell proliferation [31, 32].

To understand if using large-area low-level lasers exerted a positive effect on cell differentiation, we used immunofluorescent staining and western blot analysis to evaluate whether these lasers were capable of accelerating the induction of cell differentiation [33]. The immunofluorescent staining results showed that the nestin level in the group with i-ADSCs treated using large-area low-level laser irradiation increased substantially compared with that of the group that did not receive laser treatment. This result indicated that large-area low-level laser can accelerate the differentiation of ADSCs into neuronal cells [34, 35]. LLLT has been demonstrated to regulate neuronal function both in vitro and in vivo. Previous studies have reported that laser treatments accelerated nerve cell sprouting and cell migration, which begin within 24 h of seeding. During the first week of cultivation, irradiated cultures contain a high number of neurons exhibiting large perikaria and branched neuronal fibers, which interconnect to form networks [36]. The possible mechanism of LLLT at the cellular level has been attributed to the acceleration of electron transfer reactions, resulting in the increase of reactive oxygen species and Ca^2+^ as versatile second messengers [37]. Previous studies have shown that applying LLLT could influence cellular processes by altering DNA synthesis and protein expression [38], biomodulating cytoskeletal organization [39], and stimulating cellular proliferation [38]. Such properties suggest that LLLT, or interventions with similar neurobiological effects, can be used to treat neurodegeneration, a phenomenon that underlies debilitating clinical conditions.

However, no obvious differences were observed for GFAP and DCX in the test results. This can be explained by the differentiation agent used in this study, which exerts its effects primarily by inducing ADSCs to differentiate into NSCs. Therefore, although the ADSCs were treated using a low-level laser, they still did not differentiate into neural glia cells. The DCX protein can only be discovered after neurons have been formed from NSCs, which demonstrates that DCX is a late-stage protein that cannot be expressed when cells still exhibit the morphology of NSCs. Therefore, the amount of DCX expression is not affected by low-level laser exposure. Western blot analysis was used to determine the amount of nestin expression after i-ADSCs were exposed to large-area low-level laser irradiation and culture for 7 days. The results showed that the amount of nestin was higher with laser treatment. This is similar to the findings from the immunofluorescent-staining method, indicating that laser irradiation can accelerate the differentiation of ADSCs into neuronal cells. These results indicated that ADSCs can be induced to differentiate into neuronal cells after treatment by large-area laser irradiation for 10 min. Future studies should establish the precise duration of large-area LLLT required to achieve improved results.

In this study, treadmill and forepaws grip tests were used to evaluate motor function recovery after stem cell transplantation treatments in rats with stroke. The treadmill test results showed that the running function was weaker for the rats in the sham group on Day 7 after treatment. We speculate that this might have been because the brain-tissue lesion area from ischemia was too large; therefore, the transplanted stem cells did not have sufficient time to noticeably repair brain tissue. Therefore, the motor functions of the rats remained impaired on Day 7. When tested on Day 14, recoveries in motor functions were observed in both the i-ADSCs (LS−) and i-ADSCs (LS+) groups, with superior recovery in the i-ADSCs (LS+) group, indicating that the damaged brain tissue was repaired. On Day 21, the recovery of the rats' running function was more satisfactory in the i-ADSCs (LS+) group than in the i-ADSCs (LS−) group, indicating that the brain tissue repair capability was superior to that in the i-ADSCs (LS+) group. When tested on Day 28, the running function of the rats in the i-ADSCs (LS+) group was close to that of the rats in the sham group. However, the motor function of the i-ADSCs (LS−) group remained impaired, indicating that hind-paw motor function recovery was accelerated after i-ADSCs (LS+) treatment.

The grip test results were similar to the treadmill test results. On Day 7 after stem-cell transplantation treatment of the rats with stroke, grip strength was low for both the i-ADSCs (LS+) group and i-ADSCs (LS−) groups because the damaged brain tissues were just about to be repaired; therefore, the forepaws of the rats remained weak. When tested on Day 14, no major improvement was observed in the grip strength of the rats in the i-ADSCs (LS−) group. By contrast, the grip strength improved considerably in the i-ADSCs (LS+) group, indicating that the brain tissue repair capability was more satisfactory in the i-ADSCs (LS+) group than in the i-ADSCs (LS−) group. On Days 21 and 28, the tests showed that grip strength recovery was more satisfactory in the i-ADSCs (LS+) group than in the i-ADSCs (LS−) group. On Day 28, the grip strength in the i-ADSCs (LS+) group recovered to a level close to that of the sham group, indicating that, after i-ADSCs (LS+) treatment, the damaged brain tissues of the rats with stroke were repaired quickly, enabling the recovery of forepaw-grip strength. Based on these results, we concluded that damaged brain tissues can be repaired faster and motor function can be recovered efficiently in rats with stroke after i-ADSCs (LS+) treatment.

The i-ADSCs could differentiate into neuronal cells after transplantation into the brain. As a result, they moved and repaired damaged cerebral tissue selectively and improved cerebral functions by enhancing angiogenesis, renewal of neurons, and proliferation of nerve cells [40, 41]. In this study, we used the HE-immunostaining method and western blot analysis to evaluate brain tissue repair on Day 28 after stem cell transplantation treatment was performed in rats with stroke. The results of immunostaining were observed using a microscope. The structures were magnified 200 times, which showed that the brain tissue in the stroke lesion was dense and similar to that of normal brain tissue in the i-ADSCs (LS+) group. By contrast, numerous cavities were observed in the ischemic lesions of the brain tissues of the rats in the i-ADSCs (LS−) group. These results indicated that necrotizing brain tissue after ischemia was quickly repaired when i-ADSCs (LS+) was used to treat the rats with stroke. We speculate that using i-ADSCs (LS+) treatment can accelerate the induction of ADSCs to differentiate into neuronal cells. Therefore, although the same number of stem cells was transplanted for treatment, a greater number of NSCs were observed in the i-ADSCs (LS+) group than in the i-ADSCs (LS−) group. Furthermore, because NSCs can protect damaged brain tissues from continuous deterioration, they can also help brain tissues to repair. Therefore, transplanting a greater amount of NSCs would likely assist in repairing brain tissues more effectively.

Regarding the results of the western blot analysis, the oligo-2 amounts after stem-cell transplantation treatment were analyzed. Because oligodendrocytes form myelin in the central nervous system, myelin levels collapse when oligodendrocytes die. Nerve conduction is delayed or interrupted after the death of oligodendrocytes, leading to limb disabilities. Therefore, the evaluation of oligo-2 could be a crucial reference in assessing the degree of brain tissue recovery after stem-cell transplantation treatments in rats with stroke. In this study, using western blot analysis showed that the amount of expression of oligo-2 in brain tissues treated with i-ADSCs (LS+) was similar to that in normal brain tissues. By contrast, brain tissues treated with i-ADSCs (LS−) exhibited a lower oligo-2 expression, indicating that using i-ADSCs (LS+) treatment in rats with stroke can repair myelin in the central nervous system, leading to the recovery of neural-signal conduction and motor function. Based on these experimental results, we concluded that using i-ADSCs (LS+) treatment in rats with stroke cannot only accelerate the repair of damaged brain tissues for the partial recovery of motor functions, but also enable the central nervous system to recover the velocity of neural-signal conduction. These results confirm that the transplantation of i-ADSCs (LS+) can accelerate repairs in rats with ischemic stroke because i-ADSCs (LS+) can more efficiently differentiate into NSCs.

## 5. Conclusion

In this study, we used i-ADSCs treated with large-area low-level laser irradiation to evaluate the effects of a low-level laser on cell proliferation and differentiation. The results showed that although a low-level laser cannot facilitate cell proliferation, it can accelerate the induction of ADSCs differentiating into NSCs. In this study, we successfully created large-area cell and tissue damage in rat brains by using an embolic stroke animal model. Stem-cell transplantation with either i-ADSCs (LS+) or i-ADSCs (LS−) was performed to evaluate the degree of repair after stroke in the animals. Because large-area low-level laser irradiation can accelerate the differentiation of ADSCs into NSCs, and NSCs can protect damaged brain tissues to prevent continuous deterioration from damage and to help with repair, the motor function recovery was thus superior in the rats treated using i-ADSCs (LS+) compared with that in the rats treated using i-ADSCs (LS−). From the brain tissue slices from each group of rats, we discovered that i-ADSCs (LS+) treatment more effectively repaired necrotizing brain tissues after ischemia in rat brains. Furthermore, the western blot analysis also showed that the amount of oligo-2 increased in i-ADSCs (LS+)-treated rats with stroke, confirming the repair of myelin in cerebral neurons to further assist in the recovery of neural-signal conduction in the central nervous system.

Therefore, in the present study we demonstrated that using large-area low-level lasers exerts positive effects on inducing ADSCs differentiation, and it effectively treated ischemic stroke in rats, regarding motor function recovery. In future studies, the effects of large-area low-level laser irradiation time and the appropriate dosage for the proliferation and differentiation of ADSCs should be evaluated. If the optimal irradiation time and dosage for ADSC proliferation and differentiation can be discovered in animal experiments similar to those in this study, we believe that superior experimental results can be obtained. Furthermore, if primate or canine experimental animals can be used to conduct the experimental protocols described herein, the concerns associated with individual animal differences and errors associated with motor function assessments can be minimized to obtain more reliable experimental data. Therefore, the findings of this study contribute to the development of cell therapy, which can benefit patients with stroke.
